# Local form interference in biological motion perception

**DOI:** 10.3758/s13414-016-1092-9

**Published:** 2016-03-25

**Authors:** Jess E. Kerr-Gaffney, Amelia R. Hunt, Karin S. Pilz

**Affiliations:** School of Psychology, University of Aberdeen, William Guild Building, AB24 3FX Aberdeen, Scotland UK

**Keywords:** Motion, Biological, Object Recognition

## Abstract

Replacing the local dots of point-light walkers with complex images leads to significant detriments to performance in biological motion detection and discrimination tasks. This detriment has previously been shown to be larger when the local elements match the global shape in object category and facing direction. In contrast, studies using Navon stimuli have demonstrated that local interference on global processing primarily occurs when local elements are dissimilar to the global form. In 3 experiments, we investigated this contradiction by replacing the local dots of a point-light walker with human images or stick figures. Participants were significantly faster and more accurate at discriminating the facing and walking direction of a walker when the local images were facing in the same direction as the global walker than when they were facing in the opposite direction. These results provide support for the idea that organization of biological motion depends on allocation of limited processing resources to the global motion information when the local elements are complex. However, there is more disruption to global form processing when the local elements and global form conflict in task-related properties.

The ability to perceive biological motion, that is, the movement patterns made by humans and other animals, is crucial not only to survival but also to successful social behavior and nonverbal communication (e.g., Blake & Shiffrar, 2007; Pavlova, 2011). Biological motion is typically investigated using point-light walkers (Johansson, 1973), whereby human movement is represented by a few points attached to the major joints of the human body. Extensive research using point-light walkers has demonstrated that despite the limited information available, observers are extremely efficient in recognizing and making inferences about such displays. For example, it is possible to identify emotions (Clarke, Bradshaw, Field, Hampson, & Rose, 2005; Dittrich, Troscianko, Lea, & Morgan, 1996; Roether, Omlor, Christensen, & Giese, 2009; Spencer, Sekuler, Bennett, Giese, & Pilz, 2016), actions (Dittrich, 1993), and social characteristics, such as sex (Kozlowski & Cutting, 1977) and identity (Troje, Westhoff, & Lavrov, 2005), from point-light walkers. Recognition is also robust against noise masking (e.g., Bertenthal & Pinto, 1994). Given that point-light walkers contain little information about the form of the body, biological motion perception was originally thought to rely on the local information gained from the movement of the individual points of light (Mather, Radford, & West, 1992; Neri, Morrone, & Burr, 1998). However, patient studies (McLeod, Dittrich, Driver, Perrett, & Zihl, 1996; Vaina, Lemay, Bienfang, Choi, & Nakayama, 1990) and various experimental paradigms and stimulus manipulations in which the local motion information of the walker was degraded have demonstrated that it is possible to identify and discriminate walkers using primarily global form information (Beintema, Georg, & Lappe, 2006; Beintema & Lappe, 2002; Pilz, Bennett, & Sekuler, 2010). Nowadays, the most prevalent view is that both local motion and global form can be used to discriminate point-light walkers and that the task and stimulus used in an experiment strongly affect the relevance of the available information (e.g., Thirkettle, Benton, Scott-Samuel, 2009; Thompson & Baccus, 2012). Computational models of biological motion take into account the processing of relevant motion and form information (e.g., Giese & Poggio, 2003; Lange & Lappe, 2006). Lange and Lappe (2006), for example, proposed a two-stage model in which the first stage involved analysis of the spatial structure of the stimulus, and the second stage involved analysis of the temporal arrangement of the body templates. They suggested that the discrimination of the facing direction of point-light walkers required only the first stage, whereas walking direction discrimination (forward/backward walking) required the sequential analysis performed in the second stage.

In support of this motion-from-form model, Wittinghofer, de Lussanet, and Lappe (2010) hypothesized that biological motion perception shares processing networks with other form-related tasks, such as object recognition. They suggested that a simultaneous form-processing task should interfere with biological motion perception. Based on the findings of Hunt and Halper (2008), who demonstrated that naïve observers were unable to recognize walkers as human forms when they were composed of complex objects instead of the usual dots. Wittinghofer et al. (2010) investigated the effect of different object categories on biological motion perception. They replaced the dots of the walker with different object categories, such as vegetables, animals, or humans, and found that detection of walkers made up of human images was significantly poorer than detection of walkers made up of other object categories. Thus, the competing task of perceiving form information of the local figures can impair the integration of the individual elements into a coherent global form, especially when the local elements fall within a similar object category as the global shape. This effect is independent of the observer’s awareness of the local objects being distractors, indicating that the processing of the local form is automatic. Interestingly, when the local human images were inverted, interference was reduced, providing further evidence that the local interference is due specifically to form processing of body shape, as other visual features, as well as the motion of the images, are the same whether upright or inverted.

Extending their previous findings on object categories, Wittinghofer, de Lussanet, and Lappe (2012) proposed that the strength of interference depends on the similarity between the local objects and the global walker, and therefore, local figures facing in the same direction as the global walker should produce more interference than local figures facing in the opposite direction as the walker. In their study, point-light walkers, which were facing left or right and walking backward or forward, were composed of stick figures, also facing left or right. The task required participants to indicate the facing direction (Experiment 1) or the walking direction (Experiment 2) of the global walker as quickly as possible. As predicted, when local figures were facing in the same direction as the global walker, reaction times were slower than when the figures faced in the opposite direction; however, this effect was only true for facing-direction tasks. Wittinghofer et al. (2012) concluded that these results support an orientation-specific interference effect, whereby objects that are most similar to the walker produce the largest impediment to performance due to shared processing mechanisms.

Although consistent with the motion-from-form model, the results of Wittinghofer et al. (2012) are surprising when considered in the larger context of global and local form processing. The point-light walkers used by Wittinghofer et al. (2010, 2012) can be seen as special types of Navon (1977) stimuli: a large (global) shape made up of smaller (local) shapes. Paradigms using these compound stimuli consistently find facilitation (i.e., shorter reaction times) when the identity of the local and global shapes is congruent (e.g., a large letter H made up of small letter Hs), compared to when the identity is incongruent (Müller-Oehring, Schulte, Raassi, Pfefferbaum, & Sullivan, 2007; Pomerantz, 1983). Somewhat similar effects can be seen in Stroop tests (Stroop, 1935), whereby participants attend to one stimulus property while ignoring another. When the stimulus features are congruent, participants respond faster than when then stimulus features are incongruent (e.g., Wuhr, 2007). These effects are exactly opposite of the findings of Wittinghofer et al. (2012), who found interference (i.e., longer reaction times) when local and global object categories were the same. One explanation for this apparent contradiction is that for some tasks and object categories, congruence across levels leads to facilitation while for others it leads to interference. For example, there may be differences at the response selection and stimulus selection stages between different objects and tasks. Similar local and global elements in the Navon task might enhance response priming, leading to faster responses on congruent trials. For point-light walkers, perhaps the effects of interference at stimulus selection stages is stronger than the effects of response priming, leading to faster responses when local and global elements are dissimilar.

However, it is also possible that the different pattern of interference effects found by Wittinghofer et al. (2012) is due in part to how these effects interact with walking direction. Wittinghofer et al. (2012) used both forward- and backward-walking walkers; however, the effects sizes are relatively small, and the results are collapsed across conditions. Given that we are more familiar with people walking forward than backward, processing of backward-walking stimuli is likely to differ from processing forward-walking stimuli, and the effects of local interference might differently affect forward- and backward-walking walkers.

Here we report three experiments that further investigate the local to global interference effects for biological motion processing. In Experiment 1, we investigated the effect of local figure-facing direction on discrimination of the walking direction of the global walker, using small images of people. In this experiment, global walkers only walked forward, and participants were asked to discriminate the walking direction (left/right). If biological motion interference effects are consistent with Navon findings, participants should be faster and more accurate in their responses to walkers with local images facing in the same direction than to those with local images facing in the opposite direction. Experiment 2 was a more direct replication of Wittinghofer et al.’s (2012) first experiment, using stick figures and both forward- and backward-walking walkers. Participants were asked to discriminate the facing direction. We analyzed the results using walking direction as an additional factor and expected participants to be (a) faster and more accurate in their responses to walkers with local figures facing in the same direction than those with local figures facing in the opposite direction, and (b) faster and more accurate in their responses to walkers that walked forward than to those that walked backward. Finally, as a control, Experiment 3 assessed the effect of walking direction on facing-direction discrimination in the absence of local form interference and thus used classic point-light walkers that were walking forward or backward, and faced left or right. Again, we expected participants to be faster at discriminating the facing direction of forward- rather than backward-walking stimuli. We also expected participants to be faster overall in Experiment 3 compared to Experiment 2, confirming that local form information in general interferes with global form processing.

## Experiment 1

### Method

#### Participants

Thirty-three participants (10 male) consented to take part in the experiment. Age ranged from 19 to 33 years (*M* = 21.3, *SD* = 2.4), and all participants had normal or corrected-to-normal vision (assessed prior to the experiment using the ETDRS Logarithmic Visual Acuity chart) and gave written informed consent. The study was granted ethical approval from the Psychology Ethics Committee at the University of Aberdeen. All participants were recruited from the Aberdeen community via word of mouth and were mostly students.

#### Stimuli

**Fig. 1:**
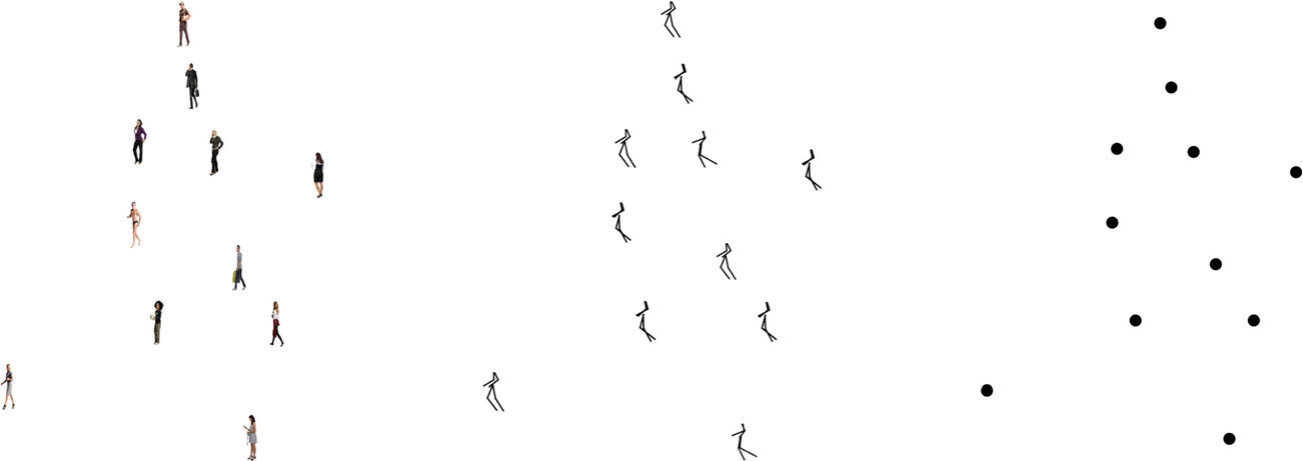
Example stimuli of all three experiments. All walkers are rightward facing with leftward-facing local images of human figures (left, Experiment 1), leftward-facing stick figures (middle, Experiment 2), or simple points (right, Experiment 3)

Point-light walkers (Vanrie & Verfaillie, 2004) consisted of 11 small images of human figures positioned on the major joints of the walker, replacing the usual point-light dots (see Fig. 1). The global walkers were positioned in the center of the computer screen while walking and facing leftward or rightward, as if on a treadmill. One walk cycle (two steps) was 40 frames long. Each frame was presented for a duration of 40 ms, resulting in a total of 1.6 s per walk cycle. The local images subtended 0.67 × 1.52 degrees of visual angle, could face left or right, and could therefore be either congruent or incongruent with the walking and facing direction of the global walker. Global walkers were presented on a white background and subtended 10 × 14.7 degrees of visual angle. Global walkers always walked forward, so the facing direction and walking direction were congruent.

#### Apparatus

Stimuli were presented using a 17-in. Viglen VL950T CRT monitor with a refresh rate of 100 Hz and a resolution of 1,024 × 786 pixels. Stimuli were generated on an Apple Mac Mini (OS X) computer using the VideoToolbox and PsychToolbox extensions for MATLAB (Brainard, 1997; Pelli, 1997; Kleiner, Brainard, & Pelli, 2007). The stimuli were viewed binocularly at a distance of 60 cm from the screen while the participant sat in an adjustable chair in a darkened room. Responses were recorded using a standard QWERTY keyboard.

#### Procedure

Participants were asked to indicate the walking direction of the global walker by pressing the X (left) or M (right) key on the computer keyboard, and they were instructed to do so as quickly and as accurately as possible. The start phases of the stimuli were randomly selected from the walking cycle on each trial, and each stimulus was shown until the participant responded, but no longer than 3.2 s (80 frames). If the participant did not respond within the given time, a screen was presented, reminding participants of the keys corresponding to the response options. After each trial, a blank screen was presented for a randomly selected time interval between 1.4 s and 2.2 s to prevent anticipation of stimulus onset.

Eighty practice trials using classic point-light walkers were presented prior to the experimental trials to familiarize participants with the task. The experimental trials comprised 80 repetitions of each of the two stimulus conditions (local figures: same or different facing direction), resulting in 160 trials in total. The walking direction of the global walker was counterbalanced across trials. Trial order was randomized for each participant individually. The experiment lasted around 30 minutes.

#### Data analysis

Paired-samples *t* tests were used to compare inverse reaction time (RT^-1^) and accuracy (% correct) across conditions. Because RTs were not normally distributed, we analyzed RT^-1^ similar to Wittinghofer et al. (2012). Figures, however, display median RTs to facilitate understanding.

### Results

#### Inverse reaction times

**Fig. 2:**
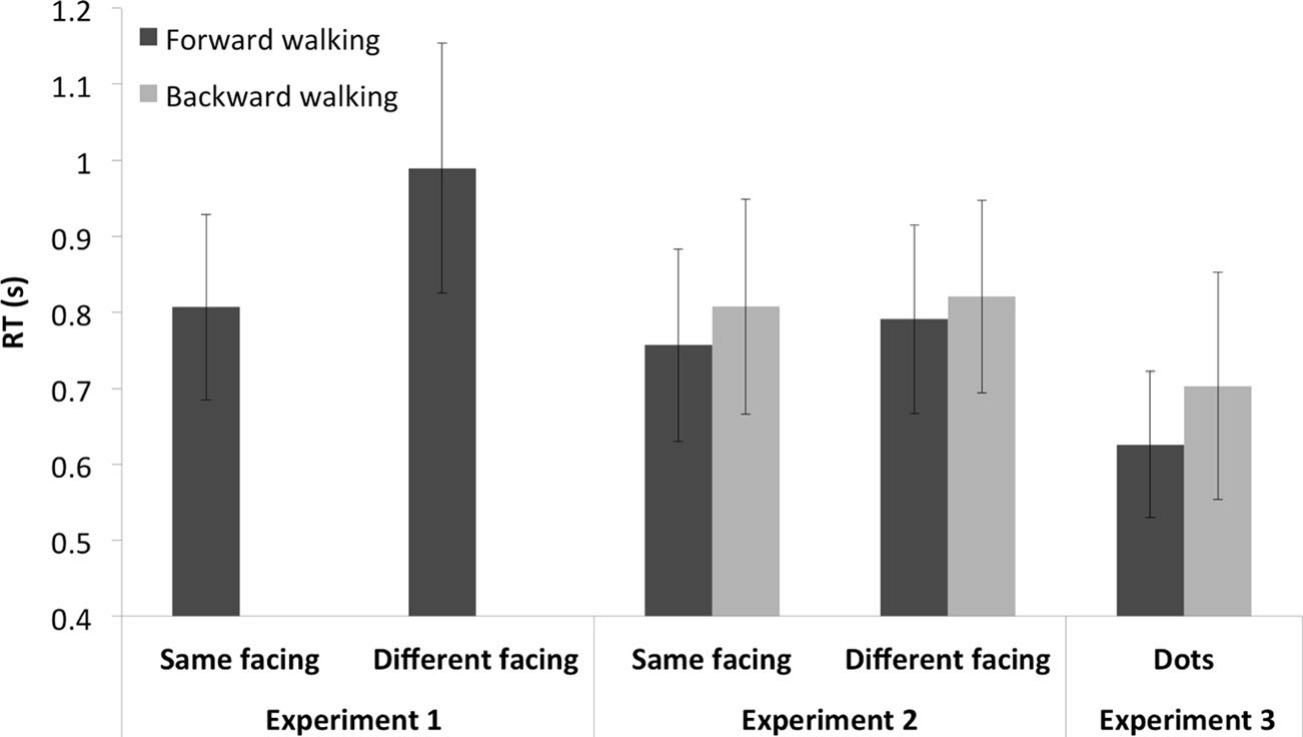
Mean reaction times for all three experiments for local images facing in the same or different direction to the global walker (Experiments 1 and 2) and walkers without local image information (Experiment 3). Error bars represent 95 % confidence intervals

Participants were significantly faster to respond to trials in which the local images were facing in the same direction as the global walker, *t*(32) = 11.5, *p* < .01; see Table 1 and Fig. 2.

**Table 1:** Mean inverse reaction time (RT^-1^) and mean of the median reaction times (RT) with standard deviations (SD) for walkers with local images facing in the same versus different direction to the global walker

	Facing direction congruence	
	Same	Different
RT^-1^ (*SD*)	1.4 (0.37)	1.18 (0.37)
RT (*SD*)	0.81 (0.36)	0.99 (0.48)

#### Accuracy

**Fig. 3:**
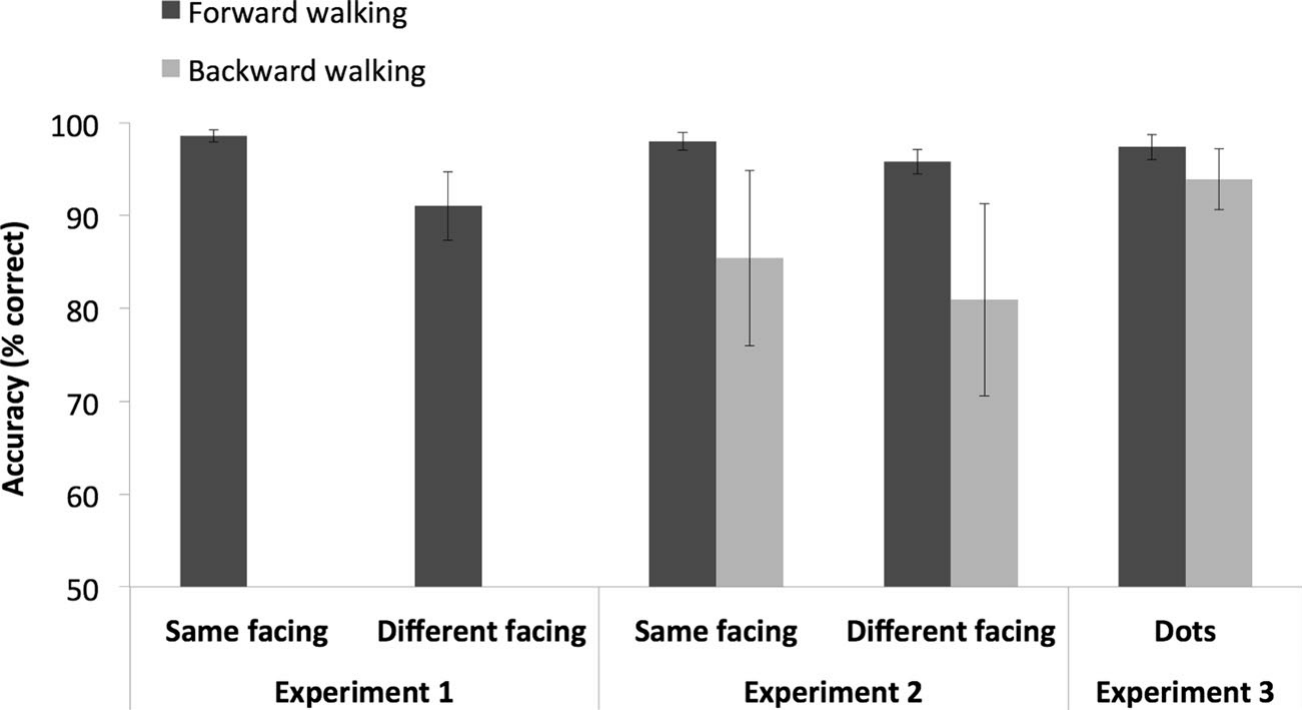
Mean accuracy for all three experiments for local images facing in the same or different direction to the global walker (Experiments 2 and 3) and walkers without local image information (Experiment 3). Error bars represent 95 % confidence intervals

Participants were significantly more accurate in identifying the walking direction of the global walker when the local images were facing in the same direction as the walker (*M* = 98.6 % ±0.66) than when they were facing in the opposite direction (*M* = 91.02 % ±3.68), *t*(32) = 5.0, *p* < .001. Mean accuracy data are shown in Fig. 3.

### Discussion

Consistent with several previous studies in global and local interactions (Müller-Oehring et al., 2007; Pomerantz, 1983), the results show that observers are faster and more accurate at discriminating the walking direction of a walker when the local images are facing in the same direction as the global figure. In contrast to the results of Wittinghofer et al. (2012), these findings suggest that while perception of point-light walkers is affected by changes on the local level, interference is stronger when local form information is in conflict with the global form. This result suggests neither the class of stimulus (people) nor the task (discriminating walking direction) can account for the discrepancy between studies using Navon-type stimuli and Wittinghofer et al. (2012). However, there were several differences between this experiment and those of Wittinghofer et al. (2012) that could have influenced the results. One potentially important difference is that we used photographic local elements, which arguably differ from the global figure more than the stick figures used by Wittinghofer et al. (2012). The dissimilarity of global and local elements may reduce interference between them, resulting in enhanced response priming effects. It may also be important that we included only forward-walking walking motion; perhaps the reduced variability in the stimulus made selection of the response-relevant information easier, thereby reducing the opportunity for local information to interfere in selection. Experiment 2, therefore, replicates their experiment more closely.

## Experiment 2

### Method

#### Participants

Fifteen participants (five male) consented to take part in the experiment. Ages ranged from 19 to 25 years (*M* = 21.8, *SD* = 1.86), and all participants had normal or corrected-to-normal vision (assessed prior to the experiment using the ETDRS Logarithmic Visual Acuity chart). All participants gave written informed consent. The study was granted ethical approval from the Psychology Ethics Committee at the University of Aberdeen. All participants were students from the university, recruited via word of mouth, and were reimbursed with a muffin.

#### Stimuli

Point-light walkers (Vanrie & Verfaillie, 2004) consisted of 11 small static stick figures positioned on the major joints of the walker, replacing the usual point-light dots (see Fig. 1, middle). The walkers were stationary in the center of the computer screen while walking backward or forward, as if on a treadmill. The local images subtended 0.67 × 1.52 degrees of visual angle, could face left or right, and could therefore be either congruent or incongruent with the facing direction of the global walker. Global walkers were presented on a white background and subtended 10 × 14.7 degrees of visual angle at the widest point of the walk cycle.

#### Apparatus

The same apparatus was used as in Experiment 1.

#### Procedure

Participants were asked to indicate the facing direction of the global walker by pressing the X (left) or M (right) key on the computer keyboard, and they were instructed to do so as quickly and as accurately as possible. The start phases of the stimuli were randomly selected from the walking cycle on each trial, and each stimulus was shown until the participant responded, but no longer than 3.2 s (80 frames). If the participant did not respond in this time, a screen was presented, reminding participants of the keys corresponding to the response options. After each trial, a blank screen was presented for a randomly selected time interval between 1.4 s and 2.2 s to prevent anticipation of stimulus onset.

Trials comprised 100 repetitions of each of the four stimuli conditions (walking direction: forward or backward, local figures: same or different facing direction), giving 400 trials in total. Trial order was randomized for each participant individually. The experiment lasted around 30 minutes.

#### Data analysis

Inverse reaction times (RT^-1^) and accuracy (% correct) for facing-direction discrimination were compared across conditions in which the local figures faced the same way as the global walkers, and those in which the local figures faced in the opposite direction to the global walker. These comparisons were analyzed separately according to whether walkers were walking backward or forward.

### Results

#### Inverse reaction times

Mean inverse reaction times were submitted to a 2 (Walking direction: forward or backward) × 2 (Local figure facing direction: same or different) repeated measures ANOVA. The analysis revealed a main effect of walking direction, *F*(1, 14) = 5.36, *p* = .036, such that participants were significantly faster to respond to walkers when they were walking forward than when they were walking backward. A main effect of local figure-facing direction was also found, *F*(1, 14) = 7.15, *p* = .018, such that participants were significantly faster to respond to walkers that were made up of local figures facing in the same direction than those in which local figures faced in the opposite direction to the walker. The interaction was not significant, *F*(1, 14) = .77, *p* = .39. Mean inverse reaction time data are displayed in Table 2. See Fig. 2 for mean reaction times.

**Table 2:** Mean inverse reaction times (RT^-1^) and mean of the median reaction times (RT) with standard deviations (SD) for backward- and forward-walking walkers with local figures facing in the same versus different direction to the global walker

	Forward		Backward	
	Same	Different	Same	Different
RT^-1^(*SD*)	1.41 (0.33)	1.34 (0.33)	1.34 (0.35)	1.29 (0.33)
RT (*SD*)	0.757 (0.25)	0.791 (0.24)	0.808 (0.28)	0.821 (0.25)

#### Accuracy

Mean accuracy was submitted to a 2 (Walking direction: forward or backward) × 2 (Local figure facing direction: same or different) repeated measures ANOVA. The analysis revealed a main effect of walking direction, *F*(1, 14) = 7.48, *p* = .016, such that participants were significantly better at discriminating the facing direction of walkers that walked forward compared to those that walked backward. The main effect of local figure facing direction was also significant, *F*(1, 14) = 11.71, *p* = .004, such that participants were better at discriminating the facing direction of walkers when the local figures were facing in the same direction compared to when they were facing in the opposite direction to the walker. The interaction was not significant, *F*(1, 14) = 3.92, *p* = .068. Fig. 3 shows that while mean accuracy was high for forward-walking trials (same = 98 % ±0.94, different = 95.8 % ±1.33), performance in backward-walking trials was considerably poorer (same = 85.4 % ±9.44, different = 80.9 % ±10.32) and more variable.

### Discussion

Consistent with the findings from Experiment 1, participants in Experiment 2 are faster and more accurate at discriminating the facing direction of point-light walkers when the local elements are facing in the same direction as the global walker than when they are facing in the opposite direction. The differences in reaction times and accuracy across conditions suggest that incongruence between local and global elements produces interference with biological motion perception, in contrast to the similarity-based interference account proposed by Wittinghofer et al. (2012). The results also confirm the prediction that participants would be better at discriminating forward-walking walkers than backward-walking walkers. Despite these performance differences, backward-walking and forward-walking walkers showed interference effects in the same general direction.

Local element walking direction did appear to have a smaller effect in Experiment 2 than in Experiment 1. Perhaps two components of global and local interactions are working in opposition here. First, and more prominently, there is a priming effect, probably operating at the response selection stage that facilitates performance when global and local elements are congruent. Second, a smaller and weaker interference effect, perhaps operating at the stimulus selection/processing stage, increases RT when local and global elements are similar (as Wittinghofer et al. observed). This latter component may have been stronger in Experiment 2, where the global and local elements were more similar, leading to smaller overall effects of congruence.

That said, overall reaction time was faster in Experiment 2 than in Experiment 1, seemingly inconsistent with the suggestion that local interference was larger in Experiment 2. However, in Experiment 1, local elements were complex and unique, two features that Hunt and Halper (2008) showed to increase interference with biological motion recognition. Multiple possible contributions to performance complicate the comparison of Experiment 1 and Experiment 2. Therefore, to confirm that local interference with global form processing was in fact operating in the context of this study, Experiment 3 provides a measure of performance with classic point-light walkers for direct, within-participants comparison with Experiment 2.

## Experiment 3

### Method

#### Participants

Twenty-five participants consented to take part in the experiment. Fifteen participants went on to complete Experiment 2; however, two were excluded from the analysis due to misunderstanding the task. The 23 remaining participants (eight male) were between 19 and 30 years old (*M* = 22.4, *SD* = 2.66), and all had normal or corrected-to-normal vision. All participants gave written informed consent. The study was granted ethical approval from the Psychology Ethics Committee at the University of Aberdeen.

#### Stimuli

Point-light walkers (Vanrie & Verfaillie, 2004) consisted of 11 point-light dots (black dots on a white background; see Fig. 1, right) positioned on the major joints of the body. The walkers were stationary in the center of the computer screen while walking backward or forward and could face either left or right, as in Experiment 2. The global walker subtended 9.5 × 14.25 degrees visual angle at the widest point of the walk cycle.

#### Apparatus

The same apparatus was used as in Experiment 1.

#### Procedure

Participants were instructed to indicate the facing direction of the walkers as quickly and as accurately as possible. The procedure was the same as that used in Experiment 2; however, 80 trials were presented in total, half of which showed the walker walking forward and half backward, with equal numbers facing left and right.

#### Data Analysis

Accuracy (% correct), and inverse reaction times (RT^-1^) were calculated for each participant to assess facing direction discrimination for forward- versus backward-walking walkers.

### Results

#### Inverse reaction times

A paired-sample *t* test showed a significant difference between conditions, *t*(22) = 4.85, *p* < .001, such that participants were faster to respond to walkers that walked forward than those that walked backward, as shown in Table 3 and Fig. 2.

**Table 3:** Mean inverse reaction time (RT^-1^) and mean of the median reaction times (RT) with standard deviations (SD) for classic forward- and backward-walking walkers

	Walking Direction	
	Forward	Backward
RT^-1^(*SD*)	1.7 (0.4)	1.6 (0.43)
RT (*SD*)	0.626 (0.24)	0.703 (0.37)

#### Accuracy

In addition, participants were significantly more accurate in their responses to trials in which walkers were walking forward (*M* = 97.4 % ±1.36) than in those where walkers were walking backward (*M* = 93.9 % ±3.27), *t*(22) = 2.4, *p* = .025, as shown in Fig. 3.

#### Comparison of Experiment 2 and Experiment 3

We collapsed accuracy and reaction times across conditions for Experiments 2 and 3 for those participants who had participated in both experiments (*N* = 13). A paired-samples *t* test showed a significant difference for inverse reaction times between experiments, *t*(12) = 5.7, *p* < .001, such that participants were faster to respond to walkers that were made of dots (Experiment 3) than those for which the local elements were replaced by images (Experiment 2), as shown in Table 4.

**Table 4:** Mean inverse reaction time (RT^-1^) and mean of the median reaction times (RT) with standard deviations (SD) for Experiment 2 and Experiment 3 (N = 13)

	Walking Direction	
	Experiment 2	Experiment 3
RT^-1^(*SD*)	1.3 (0.34)	1.69 (0.34)
RT (*SD*)	0.82 (0.26)	0.61 (0.19)

There was no significant difference in accuracy, *t*(12) = 1.8, *p* = .08, between Experiment 2 (*M* = 89.2, *SD* = 10.2) and Experiment 3 (*M* = 95.5, *SD* = 5.6).

### Discussion

As predicted, participants were faster and more accurate in their responses to point-light walkers that walked forward than those that walked backward. Taken together with the results of Experiment 2, it can be seen that discriminating backward-walking motion is more difficult than forward-walking motion, and this is true both with and without interference from conflicting local form information. Interestingly, RTs were overall significantly faster in Experiment 3, which supports the notion that the additional local image information generally interferes with the processing of the global form (Hunt & Halper, 2008; Wittinghofer et al., 2010). Accuracy was comparable between the two experiments, suggesting the interference from local elements increases processing time but does not lead participants to make an incorrect response.

## General Discussion

In a series of experiments, we investigated the effect of local form information and walking direction on recognition of biological motion stimuli. Previous work (Hunt & Halper, 2008; Wittinghofer et al., 2010) has demonstrated that when the typical dots of point-light walkers are replaced with complex objects and, in particular, human images, recognition of biological motion stimuli is greatly impaired. This impairment has been taken as evidence that biological motion perception and object or form perception share the same processing capacities. We hypothesized that walkers composed of smaller human figures that faced in the opposite direction to the global walker would produce the most interference, similar to the incongruence effects found with Navon stimuli and Stroop tasks (Navon, 1977; Stroop, 1935). While some (Wittinghofer et al., 2012) have shown that interference is strongest when local and global figures are facing in the same direction, the results of the current investigation clearly suggest the opposite. In the first experiment, small images of humans replaced the usual dots of the walker, and both the local images and the global walker were facing left or right. Participants were asked to discriminate the walking direction (left/right), and we found significantly faster reaction times and higher accuracy when local images were facing in the same direction as the global walker. In the second experiment, walking direction (forward or backward) was added as an additional factor, and participants were asked to discriminate the facing direction of the walkers. To replicate Wittinghofer et al.’s (2012) first experiment, walkers were made up of local stick figures rather than images. In accordance with Experiment 1, we found better performance when local figures faced in the same direction as the global walker, which suggests that interference is strongest when local form information about body orientation is incongruent with that of the global form. In addition, participants were significantly poorer at discriminating backward-walking walkers than those that walked forward. In Experiment 3, this effect of walking direction was found to hold true for classic point-light walkers, where conflicting local form information was absent. Comparing Experiment 2 with Experiment 3, reaction times for point-light walkers were faster when the local elements were point lights rather than stick figures, consistent with the results from Hunt and Halper (2008) and Wittinghofer et al (2010). Increased reaction times in Experiment 2 indicate that the local image information interferes with the processing of the global form. However, accuracy was comparable between experiments, which indicates that the local image information does not interfere with the execution of the correct response.

Whereas our experiments consistently found larger interference when local information was dissimilar to the global form for both facing and walking direction tasks, Wittinghofer et al. (2012) found larger interference for when local information was similar to the global form only for facing discrimination tasks. In Experiment 1, we used human images as the local stimulus and asked participants to discriminate the walking direction whereas Wittinghofer et al. used stick figures and a facing-direction discrimination task. These differences between stimuli and task might be responsible for the differing results (Thirkettle et al., 2009). However, we did not include backward-walking stimuli and, therefore, the decision that participants had to make during the walking-direction task was very similar to that of a facing-direction task (i.e., both tasks require simple left/right decisions that can be made based on the facing direction alone). In addition, we replicated our findings in Experiment 2, in which we used stick figures, included forward- and backward-walking stimuli, and asked participants to respond to the facing direction of the point-light walkers. Therefore, contradictory results between our and Wittinghofer et al.’s results cannot be explained by differences in the task or stimulus alone. We also analyzed forward and backward walkers separately and did not find an interaction with interference effects; facing direction of local figures affected both forward- and backward-walking walkers similarly, with those facing in the opposite direction to the global walker producing the most interference. This interference persisted even though participants knew that the local images were irrelevant to the task, which suggests that the processing of the local images is automatic.

Our reaction time and accuracy analyses support the idea that both interference and priming play a role in the observed effect of same-facing over opposite-facing local images. The selection of the global image requires additional time when local elements are added to the stimulus, leading to the overall increase in reaction time for Experiments 2 (with local human forms) as compared to Experiment 3 (with local dots). That said, reaction times are also overall slower in Experiment 1 than in Experiment 2, despite the local elements being arguably less similar to the global form in the former compared to the latter. This may be due to the complexity and nonuniformity of local image information in Experiment 1, which Hunt and Halper (2008) found to also interfere with biological motion recognition.

The reaction-time advantage for same-facing versus opposite-facing local stimuli could be explained by a subsequent response priming effect in that same-facing local images prime participants to prepare a response based on the local image information and, hence, lead to faster reaction times. The accuracy data support this notion in that opposite-facing local images lead not only to longer reaction times but also to lower accuracy. The interference effect of local image information on global form decisions is not easy to evaluate based on the results provided by Wittinghofer et al., given that they did not directly compare performance for walkers composed of point lights and those composed of local images. However, reaction times from Wittinghofer et al. are overall comparable to reaction times in Experiment 3, in which no local image information was present. These overall faster reaction times might indicate that interference effects from local images were overall less pronounced, which could have affected the priming that we observed in our experiments. We also observed smaller effects of local facing direction in Experiment 2, in which the local forms matched those used by Wittinghofer et al. (2012). These local forms were arguably more similar to the global form, which could have boosted their interference with stimulus selection processes. Presuming that both stimulus and response selection effects contribute to reaction time but in opposite directions, the specific combination of stimuli and responses may tilt the balance more toward one effect or the other. This could explain how similar local and global information could be found to both impede and facilitate responses.

Despite finding opposite interference effects from Wittinghofer et al. (2012), our results on the whole converge on a similar conclusion: the recognition of the local images as human shapes and the analysis of the global form of the walker use similar form-based processing mechanisms. Taking this research even further, it would be interesting to determine whether the global form of biological motion stimuli affects task decisions on the local level. This so-called global precedence is often demonstrated with Navon stimuli (e.g., Proverbio, Minniti, & Zani, 1998; Slavin, Mattingley, Bradshaw, & Storey, 2002), and applying it to biological motion could provide insight into the effects of attention on the competing local and global recognition processes.

Our results provide further evidence that local form information interferes with the processing of facing direction of biological motion stimuli. If global form information was the primary source of information used to perform the task, there should be no interference from the local form information, which would be secondary to the task at hand. However, our results show that the processing of the local images is an automatic process that interferes with the subsequent processing of the global form of the stimulus. Our results are consistent with form-based models (e.g., Lange & Lappe, 2006) and provide compelling new evidence of reduced interference when competing local form information is congruent with the global form in biological motion perception. We have also resolved a puzzling contradiction between biological motion and the more general object perception literature in terms of interference effects between global and local levels of processing. Our results confirm that similar interference effects occur in biological motion perception, consistent with the notion that biological motion is a highly routinized and familiar instance of more general perceptual and attentional process of constructing global form information from constituent parts (e.g., Cavanagh, Labianca, & Thornton, 2001).
